# Part of the gender gap in voting for Democrats arises because a higher proportion of women than men voters are Black

**DOI:** 10.1073/pnas.2221910120

**Published:** 2023-06-12

**Authors:** Paula England, Michael Hout, Karyn Vilbig, Kevin Wells

**Affiliations:** ^a^Division of Social Science, New York University Abu Dhabi, United Arab Emirates; ^b^Department of Sociology, New York University, New York, NY 10012

**Keywords:** gender gap, racial inequality, marriage, voting, elections

## Abstract

Women voted for the Democratic candidate more than men did in each US presidential election since 1980. We show that part of the gender gap stems from the fact that a higher proportion of women than men voters are Black, and Black voters overwhelmingly choose Democratic candidates. Past research shows that Black men have especially high rates of death, incarceration, and disenfranchisement due to criminal convictions. These disparities reduce the share of men voters who are Black. We show that the gender difference in racial composition explains 24% of the gender gap in voting Democratic. The gender gap in voting Democratic is especially large among those who are never-married, and, among them, the differing racial composition of men and women voters is more impactful than in the population at large, explaining 43% of the gender gap. We consider an alternative hypothesis that income differences between single men and women explain the gender gap in voting, but our analysis leads us to reject it. Although unmarried women are poorer than unmarried men, and lower-income voters vote slightly more Democratic, the latter difference is too small for income to explain much of the gender gap in voting. In short, the large gender gap among unmarried voters is not a reflection of the lower incomes of women’s households but does reflect the fact that women voters are disproportionately Black. We used the General Social Survey as the data source for the analysis, then replicated results with the American National Election Survey data.

## Significance

Social scientists have long known about the persistent gender gap in voting, with women voting Democratic more than men, and about the much larger racial differences in voting, in which a strong majority of US Blacks vote for Democrats. We show a link between these two voting gaps; the gender gap arises in part because Black women constitute a higher percentage of women voters than Black men do of men voters. This tips women’s votes toward Democrats. Other research shows that premature death, incarceration, and disenfranchisement remove Black men from the population and/or the electorate. While social science research has documented many racial disparities, one little-recognized consequence is that they contribute to the consistent post-1980 gender gap in voting Democratic.

Discussions of contemporary US politics often mention the gender gap—the tendency for a higher percentage of women than men to vote for Democrats. This gender gap emerged in the 1980s. In the 1950s, women were more Republican than men (1). Over time, women closed that original gender gap and then moved to the left of men. These US trends were part of a nearly simultaneous realignment of genders in multiple democracies in the 1960s and 1970s. In most Western democracies, women were more conservative than men, but women started moving leftward in the late 1970s and by the mid-1990s were substantially more liberal than men (2). In the United States, as we will show, there has been a gap since 1980, but the size of the gap goes up and down. Given the lack of anything close to a monotonic trend, our focus here is not on the trends but on what explains the gender gap that is present election after election.

Using data from the General Social Survey (GSS) (3) and replicating with the American National Election Surveys (ANES) (4), we show how much the difference between the % Black of men voters and women voters contributes to the gender gap in voting Democratic that has been present across all ten elections from 1980 (Reagan–Carter) to 2016 (Trump–Clinton). Then, focusing on unmarried voters, among whom the gender gap in voting is larger than among the married, we also ask whether the gap reflects the lower income of women’s households. Finding that income explains none of the gender gap; we zero in on how much gender differences in racial composition explain of the gender gap in voting Democratic among unmarried (never-married and divorced) voters.

Past research provides reasons to predict our major finding—that the higher percent Black among women than men voters explains a sizeable share of the gender gap in voting Democratic. Public health research shows that Black men have especially high rates of morbidity and death, including from homicide (5). The excess deaths of Black men mean that they are a lower proportion of the male population than Black women are of the female population. Other research highlights the uniquely high rates of incarceration for Black men (6) often keeping them from voting. Not only is it often difficult or impossible to vote while incarcerated, but some states have laws disenfranchising those with a felony conviction even years after their sentence is served (7). These factors suggest, and we will show, that male voters are disproportionately White, and women voters are disproportionately Black. This tendency of women voters to be disproportionately Black and male voters to be disproportionately White exists primarily among those who are not married, as we will also show.^*^

As mentioned above, we also examine what share, if any, of the gender gap in voting Democratic among unmarried voters is explained by single women’s households having lower income than single men’s households. One prominent view is that people typically vote their economic interests, and those with lower incomes are more likely to vote Democratic because Democrats favor more policies that redistribute to those with low incomes (12–14). Despite recent research showing that Democrats have lost the support of many White working-class voters (15), most research still shows that, on average, lower-income voters are more likely to vote Democratic than those with higher incomes (12, 14). This view suggests that the gender gap among unmarried voters might stem from women voters having lower household incomes than men voters. (The explanation would not make sense for married voters since the members of each couple share a common household income.) There is a gender gap in household income among unmarried people because most single individuals live on their own earnings, and men earn more than women (16). Moreover, if we look at household income adjusted for household size, single women’s households look even poorer because single mothers are more likely to live with their children than single fathers, and enforcement of child support laws is very imperfect (17). Thus, we investigate the extent to which, among unmarried voters, the gender gap is explained by household income differences.

## Empirical Strategy

We used the cumulative 1982 to 2018 GSS (3), which is a representative sample of English- or Spanish-speaking adults living in households.^†^ The GSS interviewed approximately 1,200 people in most years from 1982 to 1993 and then roughly 2,400 people in even-numbered years 1994 to 2018.^‡^ Our analyses included all respondents who reported having voted in the most recent presidential election (*N* = 32, 730). To replicate our analysis, we used the ANES data from 1981 to 2017 (4). These replication results are in *SI Appendix*; the ANES shows patterns similar to those in the GSS.

The dependent variable in both our GSS and the ANES analyses is the party of the candidate the respondent voted for in the last presidential election. Respondents who did not report voting in the last election were removed from the analysis. We used logistic regression to predict the dichotomous outcome of 1) voting for the Democratic candidate relative to 2) voting for someone other than the Democratic nominee (either Republican or third party). All models included dummy variables for which election the respondent was reporting on.

Our key independent variable is gender, which is coded as binary (women, men) in both the GSS and the ANES.^§^ All subsequent variables in all of our models are interacted with gender. Other control variables in all models include birth cohort and region.^¶^ We defined five cohorts: those born before 1928, 1928 to 1945, 1946 to 1965, 1966 to 1979, and 1980 to 2000. The cohorts are those used by Pew Research (19). We split the country into four regions: Northeast, Midwest, South, and West, corresponding to the US Census Bureau’s four-category classification.

Our other key independent variable is race since our main finding is that the different racial composition of women and men explains some of the gender gap. We defined four racial categories: Black of any ancestry, Hispanic of any race except Black, non-Hispanic White, and all other.^#^

To measure marital status, we classify respondents as 1) married, 2) divorced or separated (hereafter referred to as divorced), 3) never married, and 4) widowed.^‖^^,^^**^

The GSS and ANES collect income in categories. We used methods described by Hout (20) to assign a value (expressed in 1,000s of dollars) to each category and to adjust for inflation. For the GSS analysis, we adjusted for household size by dividing the income measure by the square root of the number of people in the respondent’s household, including only the respondent and persons related to the respondent (21). We then took the natural log of this measure of size-adjusted income to reflect the likelihood that proportional increases are more relevant than absolute increases (22). We used the base-2 log. (ANES does not include household size, so the ANES replication could not make the household size adjustment.)

Because it can be a sensitive question, the income variable on the GSS reduced the number of complete observations in our sample by nearly 35%. By comparison, missingness on the other variables we used never exceeded 1%. To address this issue, we imputed the missing values for this variable using Stata’s multiple imputation procedure, assuming that income was missing at random conditional on other independent variables in the model.

We performed several sensitivity tests to see whether different ways of measuring income would change our conclusion that income mediates none of the gender gap among never-married or divorced voters. First, we replaced imputed scores with the original size-adjusted income scores for those who had them, letting other cases be missing. Second, we replaced the continuous measure of size-adjusted household income with indicators for quintiles. Next, we replaced the continuous measure with untransformed household income. Next, we used the natural log of income (otherwise untransformed). Finally, instead of income, we entered three measures of socioeconomic status that are predictive of income: educational attainment, labor force participation, and whether or not the respondent had children living at home with them. We never found any of these measures of or proxies for income to mediate a nontrivial or statistically significant portion of the gender gap, as shown in *SI Appendix*, Fig. S1.

We present the results of our regression modeling in the form of graphs showing the gender marginals (the difference between the predicted percent voting Democratic for men and women) from our models.^††^*SI Appendix* contains coefficients from regression models (*SI Appendix*, Tables S5–S8) on which the figures below are based along with point estimates that are plotted in the figures (*SI Appendix*, Tables S1–S4).

Our empirical strategy depends on being able to quantify the degree to which race and income mediate the overall gender gap in voting. Doing so can be a challenge when the outcome is binary, as voting is (23). Karlson, Holm, and Breen (KHB) (24) offer tools that are very useful when exogenous variables do not interact with the more endogenous variables. But marital status is a key variable endogenous to gender (and race), and we expect and find the gender gap to vary by marital status, so we cannot use the KHB tools. Instead, we rely on marginal gender differences, which are not affected by the rescaling issue that affects logit regression coefficients (25, 26). In short, we quantify the percent explained by race by calculating the percent by which the average marginal difference between women’s and men’s vote for Democratic candidates decreases when the model is estimated with a control for race instead of without race in the model. We present the percent of the gender gap in voting Democratic explained by race for all voters, as well as within categories of marital status.^‡‡^

## Results

**Fig. 1.**
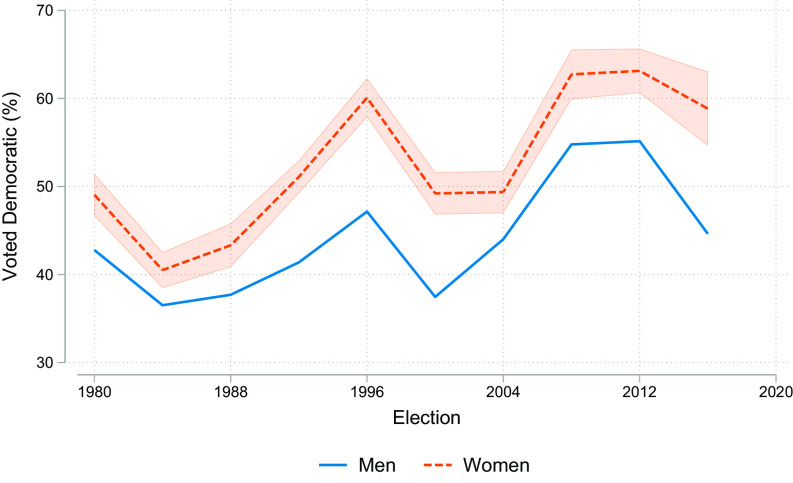
Percentage voting for the Democrat in presidential elections by election and gender, United States, 1980 to 2016. *Source:* General Social Survey, 1982 to 2018.

Fig. 1 provides percentages of women and men voting Democratic for each election from 1980 to 2016, neither smoothed nor statistically adjusted for other factors.^§§^ While the gap was at its largest in 2016, it has not steadily risen but rather goes up and down. Despite its varying size, a gender gap in which women vote more Democratic has been a fixture of American politics for nearly 40 y.

**Fig. 2.**
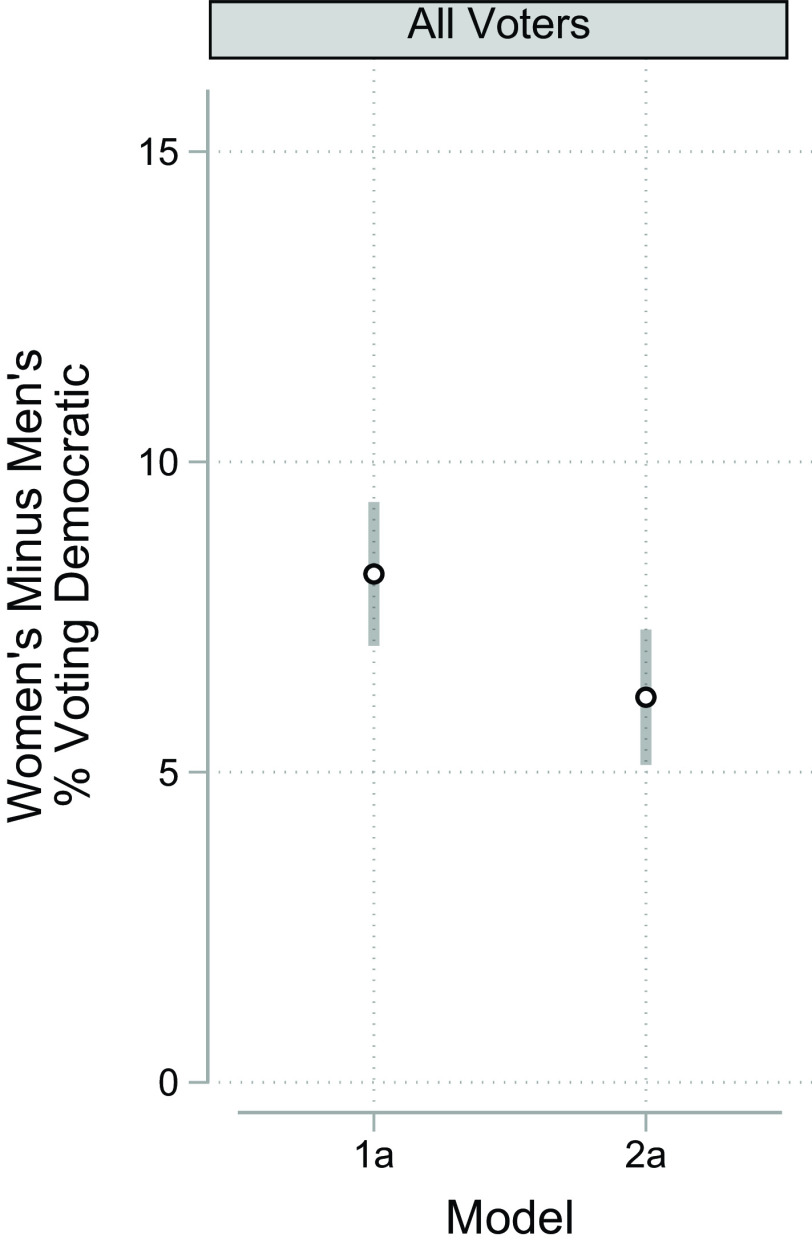
Marginal gender difference in voting for the Democrat in presidential elections by model, United States, 1980 to 2016. *Notes:***Model 1a** includes gender, cohort, and region (cohort and region are interacted with gender); **Model 2a** adds race and race*gender. *SI Appendix* for point estimates and coefficients from the underlying models. *Source:* General Social Survey, 1982 to 2018.

Fig. 2 pools elections and shows how large the gender gap is from a model that includes birth cohort and region as baseline controls. This baseline model shows an average gender gap of 8.2 percentage points, which is reduced 2.0 percentage points, to 6.2 when race is added, a drop of 24%. Thus, taking all voters in all elections together, the different racial composition of women compared to men voters explains 24% of the gender gap in voting Democratic. This stems from the relative dearth of Black voters among men voters. Our calculations show that the gender difference in racial composition of the GSS sample was substantial in the period we cover: 14% of women but just 11% of men were Black. This gap remains when considering only those members of the sample who voted: Women voters were 14% Black, while men voters were 10% Black. Given that 91% of Black voters voted for Democrats^¶¶^ compared to 40% of non-Hispanic Whites^##^ across the elections (going up and down much more among White voters), the 4 percentage point difference in the percent Black of men and women voters was very consequential. (Among Hispanics, 65% voted for the Democrat, and among those in the “all other” category, 62% voted for the Democrat. Although these two groups also voted much more Democratic than Whites, this did not explain any of the gender gap because women voters are not disproportionately from either the Hispanic or “all other” group; put another way, men and women voters do not differ in their percent who are either Hispanic or from the “all other” group.)

We next examine whether some of the gender gap is explained by women having lower size-standardized household income than men voters. Using the models described above that interact marital status with gender, we show the (model-predicted) gender gap separately for married, never-married, and divorced voters, and for each marital-status group, we show how much is explained by race and then how much of the remaining gap is explained by income.

**Fig. 3.**
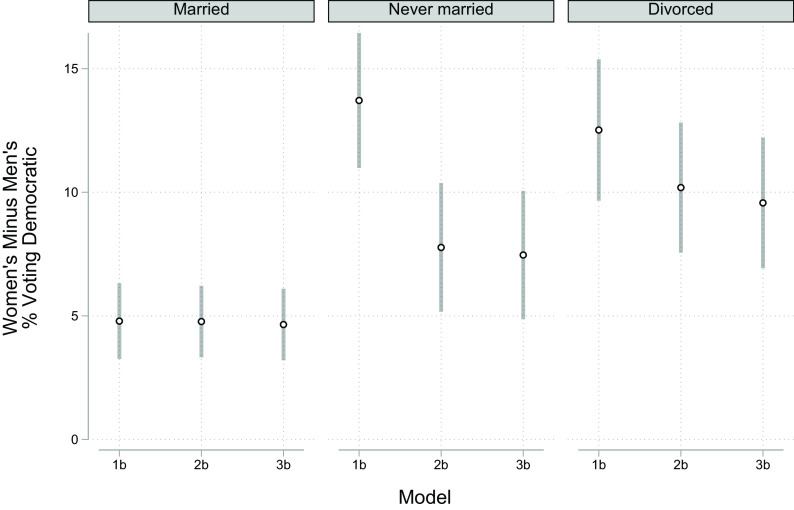
Marginal gender difference in voting for the Democrat in presidential elections by model and marital status, United States, 1980 to 2016. *Notes:***Model 1b** includes gender, cohort, and region (cohort and region are interacted with gender); **Model 2b** adds race and race*gender; **Model 3b** adds size-adjusted income and size-adjusted income*gender. See *SI Appendix* for point estimates and coefficients from the underlying models. *Source:* General Social Survey, 1982 to 2018.

Fig. 3 shows that the gender gap in voting Democratic is much larger among the two unmarried groups than among married voters. Among married voters, the gap in voting Democratic was under 5 percentage points, while among divorced voters, it was 12 percentage points, and it was 14 percentage points among never-married voters. Not only was the gap in voting Democratic modest among married voters, but race and income explained no nontrivial share of it. In the case of income, spouses sharing a common income means that there is no gender gap in income, so the null finding is expected. In the case of racial composition, there is no gender difference in the percent Black among married voters; married women were 8% Black as were 8% of married men; thus, race explained nothing.

Fig. 3 also shows that income explains none of the gender gap in voting among unmarried voters. This failure of income to mediate the gender gap among either unmarried group is perhaps surprising because computations confirm one necessary condition of mediation—that women’s households are poorer than men’s. Among never-married women voters, household income averages $45,333, whereas men’s average is $58,013, and women are poorer among divorced voters as well ($43,348 versus $60,540). The per capita income gap is even larger because 25% of never-married women voters live with children, compared to 10% among men, with a large gap among the divorced as well (39% versus 15%). Despite these differences, because the effect of income on voting was very modest, and not statistically significant for women, income explained less than one percent of the gap among divorced or never-married voters. Moreover, the conclusion that income failed to explain a significant amount of the gender gap in voting Democratic holds under several sensitivity tests described in the section on our Empirical Strategy and shown in *SI Appendix*.

**Fig. 4.**
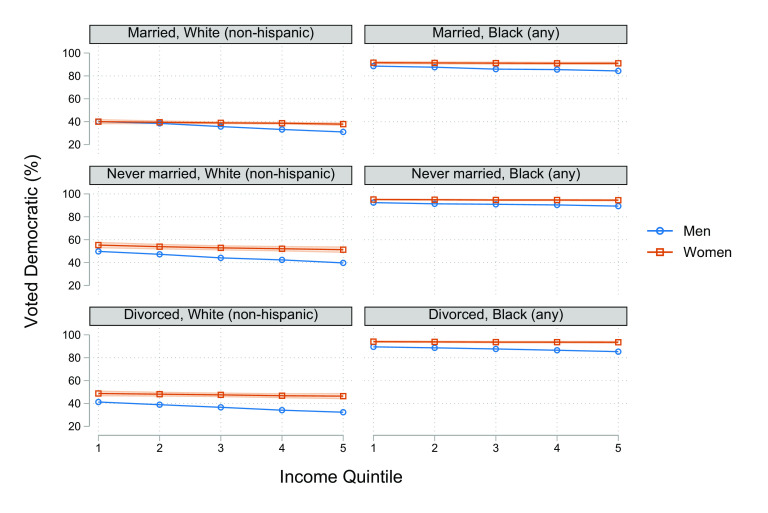
Predicted % voting Democratic across income quintiles by gender, race, and marital status, 1980 to 2016. *Notes:* Estimates based on Model 3b with the continuous income measure converted to quintiles. See *SI Appendix* for point estimates and coefficients from the underlying model. *Source:* General Social Survey, 1982 to 2018.

As an illustration of why the (negative) effect of income on voting Democratic is too small to explain any nontrivial share of the gender gap, Fig. 4 shows the percent of voters voting Democratic (pooling elections) from our sensitivity test that puts the size-standardized income measure into quintiles. It shows that, for all groups except White men, there is less than a 5 percentage point difference between the percent voting Democratic in the top and bottom quintiles. By comparison, the difference between Black and White voters is approximately 50 percentage points. Race matters immensely, but income makes only a very small difference. In sum, single women are poorer than single men, but that is not the reason they vote Democratic.

What is important in explaining the gap among unmarried voters is the gender gap in racial composition, as Fig. 3 clearly shows. For context, never-married voters make up 17% of our sample of voters, and 16% are divorced, so together, they are approximately one third of voters. Computations show that the racial composition of men and women voters differs much more in the unmarried groups than among married voters. Among married voters, the difference between the percent Black among women and men was less than one percentage point. By contrast, there was a difference of 4 percentage points among divorced voters (women were 20% Black compared to 16% among men) and an even larger 12 percentage points among never-married voters (26% of women were Black compared to 14% of men). As a consequence, the 14 percentage point gap in voting Democratic among never-married voters dropped to 8 after controlling for race (Fig. 3), a very large drop of 43%, which is also statistically significant (That is, the estimated gap from the model including race does not fall within the 0.05 two-tailed CI of the estimated gap from the model without race). Among divorced voters, controlling for race reduced the gender gap 17%, from 12 percentage points to 10 (Fig. 3), although this reduction barely escapes statistical significance.

**Fig. 5.**
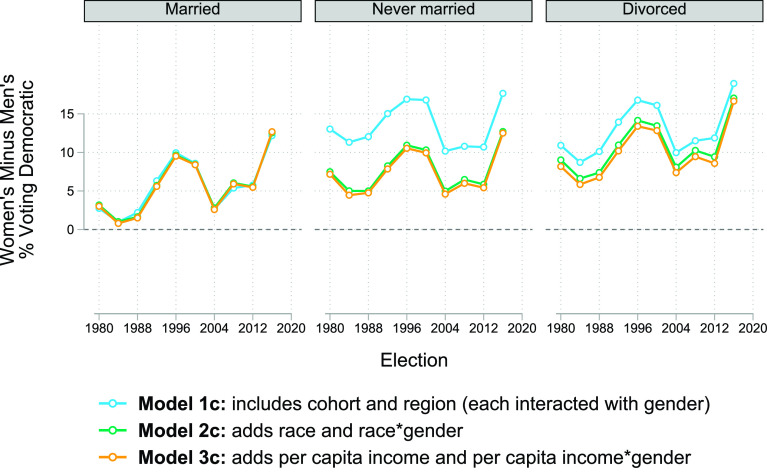
Marginal gender difference in voting for the Democrat in presidential elections by election, model and marital status, United States, 1980 to 2016. *Notes:* See *SI Appendix* for point estimates and coefficients from the underlying models. *Source:* General Social Survey, 1982 to 2018.

These conclusions—that the gender gap is larger among unmarried voters, that family income differences between women and men voters explain no nontrivial part of the gap, and that race explains a sizeable share of the gap among never-married voters—hold for every election year analyzed, as Fig. 5 shows.

## Discussion

We explored whether women vote more Democratic because a higher proportion of them are Black and whether, among unmarried voters, women vote Democratic more than men because of their lower household incomes. Regarding income, we found that, while, on average, unmarried women voters live in poorer households than unmarried men voters, this is not why they vote more Democratic. No matter how we measured income, controlling for it did not reduce the gender gap any nontrivial amount.

One key factor in the gender gap is racial composition; the different racial composition of women and men voters explains 24% of the gender gap across all elections between 1980 and 2016 combined when we pool married and unmarried voters. Our exploration further revealed that the gender gap is relatively small among married voters, larger among divorced voters, and largest among never-married voters. Race composition is unimportant in explaining what gap there is among the married, so almost the entirety of the 24% of the overall gender gap that is explained by the different racial composition of men and women comes from unmarried—divorced and never-married—voters. Indeed, among the never-married, 43% of the gender gap is explained by differences between men and women’s racial composition.

Our analysis makes clear that the gender gap in voting stems in part from racial inequalities. The uniquely high mortality, incarceration, and disenfranchisement of Black men lead to a dearth of Black men in both the population broadly and among voters specifically. This in turn means that men voters are disproportionately White and women voters are disproportionately Black. Moreover, more Black women than White women are unmarried, making the difference in racial composition of unmarried men and women voters especially large. Black women often find themselves single because of a combination of factors—the lack of Black men in the population, their incarceration rates, the proportion who may be seen as “unmarriageable” because labor market discrimination and/or incarceration has led to their joblessness (6, 27), and the higher number of Black men than women who marry interracially (28, 29). All these racial inequalities contribute to the larger gender difference in racial composition among unmarried than married voters. But, the large gender difference in the percent of unmarried voters that are Black would not create such a large gender difference in voting Democratic if Whites and Blacks did not vote so differently. Across the elections we analyzed, 1980 to 2016, 91% of votes cast by Black voters were for Democrats, compared to 40% among Whites. This too is undoubtedly a legacy of discrimination and continuing racial inequality. The most extreme case of this link between racial and gender inequalities can be seen among never-married voters; in this group, women are 26% Black and men 14% Black, and this difference in racial composition explains 43% of the gender gap.

## Supplementary Material

Appendix 01 (PDF)

## Data Availability

All data used here are publicly available at the GSS website: gss.norc.org/ (18). Code for data analysis is archived on Open Science Framework (osf.io/pvdnm/). Previously published data were used for this work (3).

## Footnotes

^*^Our analysis of race focuses on the explanatory power of the higher percent Black of women than men voters in explaining the gender gap in voting, given the strong tendency of all Blacks to vote Democratic. It is logically possible for a compositional effect in this direction to be in force whether or not there is a gender gap in voting, or in voting Democratic, within each racial group. In reality, past research shows that these gaps exist as well. First, there is a gender gap in voting among those eligible to vote, such that a lower percent of men than women vote, and this gap is larger among Blacks than Whites (8, 9). There is also a gender gap in who votes Democratic among Whites, Blacks, and other groups; the gap among Blacks had been smaller than that in other groups (10) but was the same as for Whites in 2016 (11).

^†^Response rates were very good until recently; an average of 77% of selected adults in sampled residences completed interviews 1975 to 1998, compared to 70% in 2000 to 2014 and 60% in 2016 to 2018 (3, p. 3189).

^‡^The 2021 GSS dataset isnow available, butwechose not to add it. The COVID-19 pandemic disrupted the GSS, preventing face-to-face interviews and delaying data collection until December 2020 and early 2021 (18). Until the 2022 data are available, it will be hard to apportion differences between the most recent data and the rest of the time series to changes in politics versus changes in the data collection method.

^§^Since 2016, the GSS included a nonbinary gender classification, but the only measure available for all years is binary.

^¶^Birth cohort is clearly exogenous. Region can change, but a large share of US residents live in the region in which they were born, making region exogenous for most.

^#^The GSS did not ask about Hispanic heritage explicitly until 2000. In all years of the survey, however, the GSS asks “From what countries or part of the world did your ancestors come?” Non-Black respondents who answered Mexico, Puerto Rico, Spain, West Indies, or Other Spanish were marked as Hispanic. While it is more common to assign Hispanic Blacks to the Hispanic category, we did not do this because the Hispanic classification in the GSS is less certain than the racial classification in the early years.

^‖^In a sensitivity test, we created a fourth marital status category, “remarried,” for those who have been divorced but are currently married, removing them from the married category. They were much more like the married than the divorced in having a smaller gender gap that was not much explained by any of our control variables, which supports our decision to treat all currently married persons as having the same marital status.

^**^Widows and widowers are a very small group for which estimates were, thus, much less precise. We excluded them from the figures and discussion below, but they are in the underlying statistical analyses.

^††^To obtain the gender marginal based on a given model, Stata makes three calculations for each observation in the data. First, treating the case as female, it sets the female variable to 1 and inserts that 1 plus that case’s observed values on all other variables into the regression equation to obtain a predicted “female” value on the dependent variable for each case. Second, it analogously treats the same case as male, obtaining a prediction exactly as above except for adding a 0 for the female variable to denote that the case is male. Finally, it calculates the difference between the “female” and “male” cases. These three calculations are done for each case in the dataset. The marginal gender difference for a given subgroup (for example, marital status) is the average of these “female”–“male” differences across all cases in the subgroup.

^‡‡^In *SI Appendix*, Fig. S2, we divide voters by whether they are college graduates and show how much of the gender gap (within marital status categories) is explained by race separately for college graduates and those without a college degree. It is among unmarried voters who are not college graduates that the percent Black among women voters most exceeds that among men and in which gender differences in racial composition explain most of the gender gap.

^§§^Predicted percents are from a model with gender, election year, and their interactions, which makes them essentially descriptive statistics.Wedid, however, apply design weights.

^¶¶^Among Black voters 88% of men and 93% of women voted Democratic.

^##^Among White voters 37% of men and 43% of women voted Democratic.
