# Supplementary material for: Part of the gender gap in voting for Democrats arises because a higher proportion of women than men voters are Black
Source: Proc Natl Acad Sci U S A. 2023 Jun 12;120(25):e2221910120. doi: 10.1073/pnas.2221910120 (PMC10288652; doi:10.1073/pnas.2221910120)
Supplement: Supplementary file 1 — Appendix 01 (PDF) [file pnas.2221910120.sapp.pdf]

Supporting Information for

Part of the gender gap in voting for Democrats arises because a higher proportion of women than men voters are black

Paula England, Michael Hout, Karyn Vilbig, and Kevin Wells

Paula England.
E-mail: pengland@nyu.edu

This PDF file includes:

Figs. S1 to S7
Tables S1 to S8

Introduction and Overview of SI Appendix. In this Supplementary Information (SI) Appendix, we present additional analyses in support of our conclusions. Figure S1, using GSS data, contains robustness checks for the conclusion of the paper that income differences between single men and women do not explain any appreciable share of the gender gap in which women vote more Democratic. Figure S2, using GSS data, responds to a reviewer's request that we show our analysis in Figure 3 (of the main paper) separately for those who are not college graduates; Figure S2 shows results for each of college graduates and those without a college degree, showing that racial composition explains much more of the gender gap among unmarried voters without a college degree, which we believe is supportive of our interpretation in terms of black men's incarceration and homicide death. Tables S1-S4 show the numbers underlying Figures 2, 3, 4, and 5 in the paper, using GSS data. Tables S6-S8 provide the coefficients underlying the models graphed in Figures 3-5 in the paper. The remaining figures show our replications with the American National Election Survey; Fgures S3-S7 parallel the figures shown in the paper from GSS data.

Robustness Checks of Income's Non-mediation. Figures 3 and 5 in the main paper showed that controlling for income explains none of the gender gap for voters of any marital status. We undertook number of sensitivity tests, summarized in Figure S1, to see if measuring income in a different way would change this conclusion. Models 2b and 3b reproduce models in Figure 3 in the main paper. Then as an alternative to Model 3b (which adds log base 2 size-standardized income, with missing income values obtained through imputation), Model A1 treats missing cases as missing instead of imputing them. No significant mediation of the gender effect is seen. (The remaining sensitivity tests also do not impute for missing values.) Model A2 relaxes the assumption that income's effect is linear, adding indicators for quintiles of the measure in Model 3b, while Model A3 uses household income with no transformation or standardization, and Model A4 uses the natural log of income with no other transformation. Model A5 controls for determinants of size-adjusted income instead of income itself; these determinants are education, labor force participation, and presence of children. In none of these cases was any nontrivial or statistically significant amount of the gender gap explained by income. This buttresses our conclusion that while single women are poorer than single men, that is not the reason they vote more Democratic.

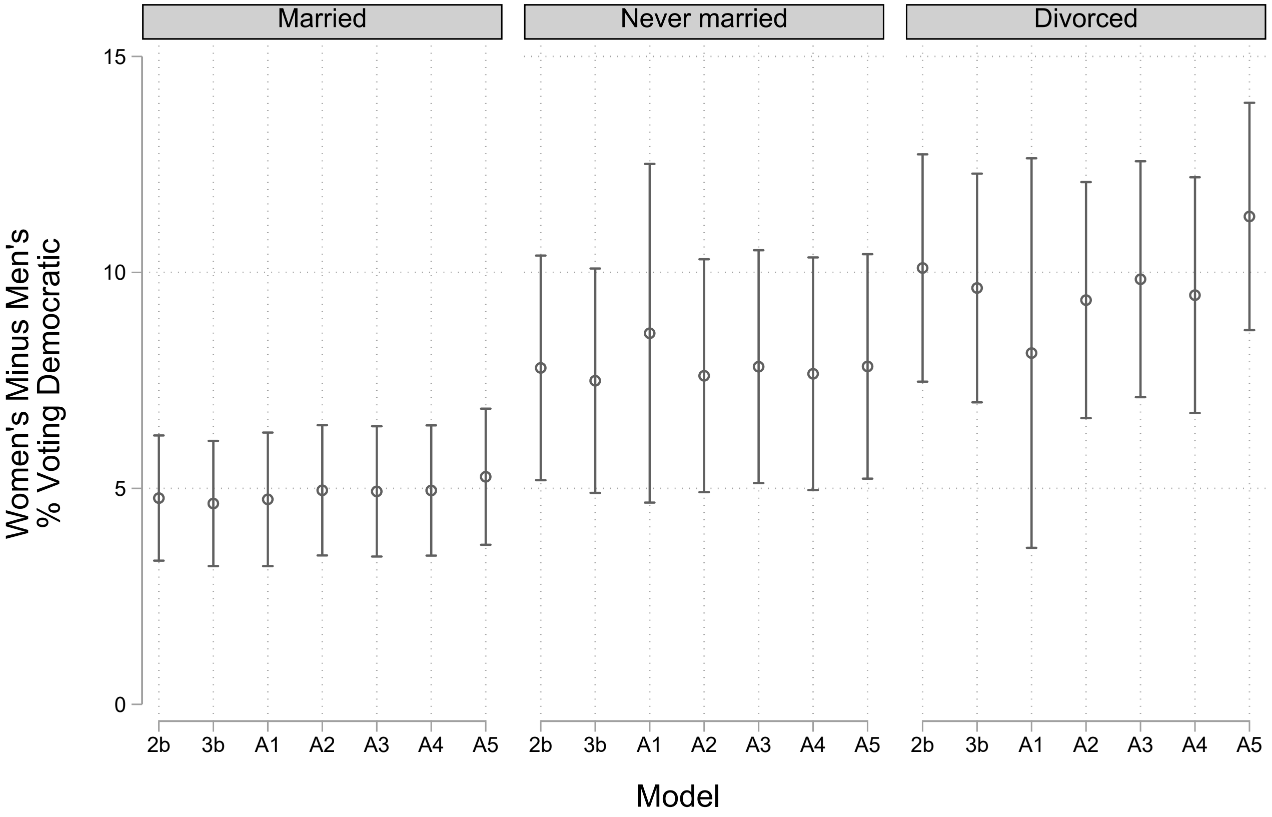

Fig. S1. Marginal Gender Difference in Voting Democratic in Presidential Elections, by Marital Status, Under 7 Models to Explore Income Mediation, 1980-2016.
Model 2b Includes race and race*gender
Model 3b Adds size-standardized income (with imputation) and size-standardized income*gender to Model 2
Model A1 Adds size-standardized income (without imputation) and size-standardized income*gender to Model 2
Model A2 Adds income quintile and income quintile*gender to Model 2
Model A3 Adds income (no transformation) and income*gender to Model 2
Model A4 Adds logged income and logged income*gender to Model 2
Model A5 Adds educational attainment, labor force participation and the presence of children in the household (and each one's interaction with gender) to Model 2
Notes: Source: General Social Surveys, 1982-2018.

Exploring How Much Racial Composition Mediates the Gender Gap Separately by Educational Groups. Figure S2 shows that the explanatory power of racial composition is much greater for those who do not hold a college (BA/BS) degree than those with such a degree. Given that black men's high rates of incarceration or death by homicide are highest among unmarried men without college degrees, the finding in Figure S2 is consistent with our interpretation that the dearth of black men among voters arises, at least in part, because of disadvantaged black men's high rates of incarceration and homicide. The Figure shows that, among never-married voters without a college degree, race significantly mediates the gender effect. Calculations from the regressions show that, among these never-married voters without a college degree, the gender difference in racial composition explains 57% of the gender gap in voting Democratic. Among those with college degrees, race does not mediate the gender gap with either statistical significance or a nontrivial magnitude. Figure S2 also makes clear that the larger size of the gender gap found among never-married voters in the main paper's Figure 3 comes largely from the especially large gender gap among those never-married voters who do not have college degrees.

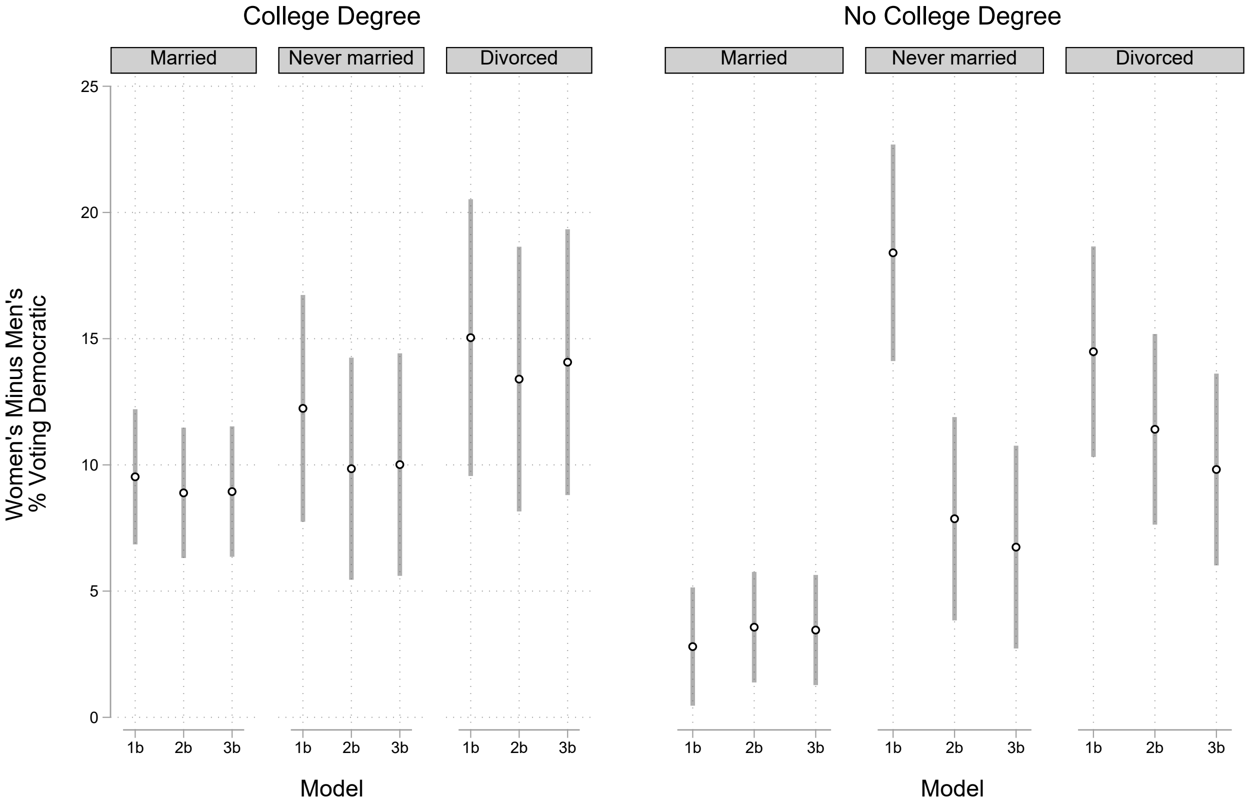

Fig. S2. Marginal Gender Difference in Voting Democratic in Presidential Elections, by Marital Status and Educational Attainment, Under 3 Models, 1980-2016.
Model 1b Includes gender, cohort, and region (cohort and region are interacted with gender)
Model 2b Adds race and race*gender
Model 3b Adds size-standardized income (with imputation) and size-standardized*gender to Model 2

Notes: Source: General Social Surveys, 1982-2018.

Numbers Underlying Figures in the Paper. Table S1, below, gives the numbers graphed in Figure 2, the AMEs of gender (i.e. the predicted gender differences) from a baseline model and from a model controlling race. It shows that the gender difference after controlling for race is substantially smaller, implying that the gender difference in racial composition explains some of the gender gap in voting Democratic. Table S2 provides the AMEs of gender that are shown graphically in Figure 3 in the paper, for each of 3 marital statuses, for each of the baseline model, the model adding race, and the model adding size-standardized income. Table S3 provides the predicted proportions voting Democratic, by marital status, gender, race, and size-standardized household income quintile; these are what are shown graphically in Figure 4 in the paper. They show that the the tendency of voters in higher income quintiles to vote less Democratic is very slight, especially for those other than white men, and illustrates why we did not find income to mediate the gender gap. Table S4 provides the AMEs of gender graphed in Figure 5 in the paper.

Table S1. Average Marginal Effects in Figure 2

Model 1a	.082
	(.006)
Model 2a	.062
	(.006)

Source: GSS 1982-2018.
Standard errors in parentheses.

Table S2. Average Marginal Effects in Figure 3

	Married	Never Married	Divorced	Widowed
Model 1b	.048	.137	.124	.002
	(.008)	(.014)	(.015)	(.023)
Model 2b	.048	.078	.101	.002
	(.007)	(.013)	(.013)	(.022)
Model 3b	.047	.075	.096	-.001
	(.007)	(.013)	(.014)	(.022)

Source: GSS 1982-2018.
Standard errors in parentheses.

Table S3. Predicted Proportions in Figure 4

	Income Quintile	Married	Never Married	Divorced
White men	1	.401<br>(.013)	.498<br>(.015)	.413<br>(.016)
	2	.385<br>(.009)	.472<br>(.013)	.389<br>(.014)
	3	.357<br>(.006)	.441<br>(.011)	.366<br>(.012)
	4	.332<br>(.006)	.423<br>(.012)	.341<br>(.012)
	5	.310<br>(.008)	.397<br>(.013)	.323<br>(.014)
White women	1	.400<br>(.012)	.553<br>(.014)	.487<br>(.013)
	2	.395<br>(.008)	.539<br>(.013)	.481<br>(.011)
	3	.390<br>(.006)	.529<br>(.012)	.475<br>(.010)
	4	.386<br>(.006)	.521<br>(.012)	.468<br>(.011)
	5	.378<br>(.008)	.513<br>(.014)	.464<br>(.014)
Black men	1	.886<br>(.010)	.924<br>(.008)	.895<br>(.010)
	2	.876<br>(.011)	.914<br>(.009)	.886<br>(.010)
	3	.859<br>(.012)	.909<br>(.009)	.876<br>(.011)
	4	.855<br>(.012)	.904<br>(.010)	.865<br>(.012)
	5	.844<br>(.013)	.893<br>(.012)	.853<br>(.014)
Black women	1	.916<br>(.007)	.951<br>(.004)	.940<br>(.005)
	2	.913<br>(.007)	.949<br>(.004)	.938<br>(.005)
	3	.912<br>(.007)	.947<br>(.005)	.936<br>(.005)
	4	.910<br>(.007)	.947<br>(.005)	.936<br>(.006)
	5	.910<br>(.007)	.945<br>(.005)	.935<br>(.006)

Source: GSS 1982-2018.

Standard errors in parentheses.

Table S4. Average Marginal Effects in Figure 5

		Married	Never Married	Divorced
1980	Model 1c	.027<br>(.019)	.130<br>(.024)	.108<br>(.024)
	Model 2c	.032<br>(.019)	.075<br>(.023)	.089<br>(.022)
	Model 3c	.030<br>(.019)	.072<br>(.023)	.082<br>(.022)
1984	Model 1c	.010<br>(.016)	.113<br>(.022)	.086<br>(.022)
	Model 2c	.010<br>(.015)	.050<br>(.021)	.065<br>(.019)
	Model 3c	.008<br>(.015)	.044<br>(.021)	.059<br>(.019)
1988	Model 1c	.022<br>(.019)	.121<br>(.024)	.100<br>(.024)
	Model 2c	.017<br>(.019)	.051<br>(.023)	.074<br>(.022)
	Model 3c	.015<br>(.019)	.048<br>(.023)	.068<br>(.022)
1992	Model 1c	.063<br>(.015)	.151<br>(.019)	.138<br>(.020)
	Model 2c	.058<br>(.014)	.083<br>(.018)	.109<br>(.018)
	Model 3c	.057<br>(.014)	.079<br>(.018)	.104<br>(.018)
1996	Model 1c	.099<br>(.018)	.169<br>(.020)	.167<br>(.021)
	Model 2c	.096<br>(.018)	.109<br>(.019)	.140<br>(.020)
	Model 3c	.095<br>(.018)	.105<br>(.019)	.134<br>(.020)
2000	Model 1c	.085<br>(.018)	.168<br>(.022)	.160<br>(.023)
	Model 2c	.084<br>(.017)	.103<br>(.020)	.133<br>(.020)
	Model 3c	.084<br>(.017)	.100<br>(.020)	.129<br>(.021)

Table S4. (continued)

		Married	Never Married	Divorced
2004	Model 1c	.027	.102	.099
		(.019)	(.024)	(.023)
	Model 2c	.028	.050	.080
		(.018)	(.022)	(.021)
	Model 3c	.026	.046	.074
		(.018)	(.022)	(.021)
2008	Model 1c	.054	.108	.114
		(.025)	(.024)	(.026)
	Model 2c	.061	.065	.102
		(.024)	(.022)	(.024)
	Model 3c	.059	.060	.095
		(.024)	(.022)	(.024)
2012	Model 1c	.057	.107	.118
		(.021)	(.022)	(.023)
	Model 2c	.056	.059	.094
		(.020)	(.019)	(.020)
	Model 3c	.055	.054	.086
		(.020)	(.019)	(.020)
2016	Model 1c	.122	.177	.189
		(.032)	(.034)	(.034)
	Model 2c	.125	.127	.169
		(.030)	(.030)	(.031)
	Model 3c	.127	.125	.166
		(.030)	(.030)	(.031)

Source: GSS 1982-2018.
Standard errors in parentheses.

Coefficients From Models Underlying Figures 2-5. Tables S5-S8 provide the coefficients from the models upon which Figures 2-5 in the main paper are based.

Table S5. Coefficients from the Models in Figure 2.
All models are logistic regressions that interact each variable (other than gender) with gender. Model 1a is a baseline model that controls for birth cohort and region. Model 2a adds race.

	Model 1a	Model 2a
Women	0.471***	0.419***
	(0.063)	(0.067)
Before 1928	0.218***	0.372***
	(0.054)	(0.057)
1928-1945	0.042	0.103*
	(0.046)	(0.049)
1966-1979	0.222***	0.161**
	(0.056)	(0.060)
1980-2000	0.722***	0.579***
	(0.092)	(0.099)
Before 1928 x Women	-0.396***	-0.384***
	(0.070)	(0.075)
1928-1945 x Women	-0.103	-0.082
	(0.061)	(0.065)
1966-1979 x Women	-0.027	-0.078
	(0.074)	(0.080)
1980-2000 x Women	-0.057	-0.051
	(0.123)	(0.132)
Midwest	-0.133*	-0.110
	(0.055)	(0.058)
South	-0.212***	-0.431***
	(0.053)	(0.057)
Pacific	-0.205***	-0.223***
	(0.059)	(0.061)
Midwest x Women	-0.104	-0.126
	(0.073)	(0.077)
South x Women	-0.032	-0.071
	(0.070)	(0.075)
Pacific x Women	-0.052	0.020
	(0.078)	(0.081)
Black (any)		2.650***
		(0.096)
Hispanic (non-black)		1.026***
		(0.090)
All other		0.819***
		(0.147)
Black (any) x Women		0.342**
		(0.128)
Hispanic (non-black) x Women		-0.099
		(0.119)
All other x Women		0.114
		(0.194)
Constant	-0.221***	-0.475***
	(0.048)	(0.051)
Observations	32730	32730

Source: GSS 1982-2018.
Standard errors in parentheses
* p<0.05, ** p<0.01, *** p<0.001 (two-sided tests)

Table S6. Coefficients from the Models in Figure 3.
All models are logistic regressions that interact each variable (other than gender) with gender. Model 1b is a baseline model that controls for birth cohort and region. Model 2b adds race. Model 3b adds log base 2 household income divided by the square root of the number of people living in the household.

	Model 1b	Model 2b	Model 3b
Women	0.347***	0.366***	-0.072
	(0.068)	(0.071)	(0.177)
Birth Cohort: Before 1928	0.222***	0.378***	0.318***
	(0.057)	(0.060)	(0.062)
Birth Cohort: 1928-1945	0.080	0.139**	0.130**
	(0.047)	(0.050)	(0.050)
Birth Cohort: 1966-1979	0.156**	0.092	0.088
	(0.057)	(0.061)	(0.062)
Birth Cohort: 1980-2000	0.543***	0.402***	0.389***
	(0.094)	(0.102)	(0.102)
Birth Cohort: Before 1928 x Women	-0.504***	-0.481***	-0.435***
	(0.078)	(0.082)	(0.084)
Birth Cohort: 1928-1945 x Women	-0.143*	-0.128	-0.121
	(0.063)	(0.067)	(0.067)
Birth Cohort: 1966-1979 x Women	-0.030	-0.046	-0.042
	(0.076)	(0.082)	(0.082)
Birth Cohort: 1980-2000 x Women	-0.069	0.010	0.020
	(0.128)	(0.136)	(0.136)
Region: Midwest	-0.124*	-0.100	-0.125*
	(0.056)	(0.058)	(0.059)
Region: South	-0.203***	-0.417***	-0.442***
	(0.054)	(0.057)	(0.058)
Region: Pacific	-0.205***	-0.225***	-0.242***
	(0.059)	(0.062)	(0.062)
Region: Midwest x Women	-0.090	-0.120	-0.097
	(0.074)	(0.077)	(0.078)
Region: South x Women	-0.027	-0.065	-0.044
	(0.071)	(0.076)	(0.076)
Region: Pacific x Women	-0.043	0.023	0.039
	(0.079)	(0.082)	(0.082)
Marital status: Never married	0.444***	0.400***	0.363***
	(0.051)	(0.054)	(0.055)
Marital status: Divorced	0.310***	0.147**	0.107
	(0.052)	(0.056)	(0.057)
Marital status: Widowed	0.504***	0.367***	0.323***
	(0.088)	(0.094)	(0.095)
Marital status: Never married x Women	0.328***	0.093	0.119
	(0.071)	(0.076)	(0.077)

Table S6. (continued)

	Model 1b	Model 2b	Model 3b
Marital status: Divorced x Women	0.279***	0.230**	0.257***
	(0.068)	(0.073)	(0.075)
Marital status: Widowed x Women	0.046	0.018	0.051
	(0.102)	(0.110)	(0.111)
Race: Black (any)		2.624***	2.573***
		(0.097)	(0.098)
Race: Hispanic (non-black)		1.033***	1.003***
		(0.090)	(0.090)
Race: All other		0.843***	0.846***
		(0.147)	(0.146)
Race: Black (any) x Women		0.269*	0.305*
		(0.129)	(0.130)
Race: Hispanic (non-black) x Women		-0.135	-0.119
		(0.119)	(0.120)
Race: All other x Women		0.082	0.076
		(0.194)	(0.194)
Size-adjusted household income			-0.092***
			(0.022)
Women x Size-adjusted household income			0.072**
			(0.027)
Constant	-0.365***	-0.580***	-0.026
	(0.051)	(0.054)	(0.144)
Observations	32730	32730	32709

Source: GSS 1982-2018.

Standard errors in parentheses

* p<0.05, ** p<0.01, *** p<0.001 (two-sided tests)

Table S7. Coefficients from the Model in Figure 4.
This model is like Model 3b except that it uses quintiles of size-standardized income as opposed to size-standardized income. Like Model 3b, it is a logistic regression that interacts each variable (other than gender) with gender.

	Figure 4 Model
Women	0.109
	(0.117)
Birth Cohort: Before 1928	0.318***
	(0.061)
Birth Cohort: 1928-1945	0.132**
	(0.050)
Birth Cohort: 1966-1979	0.072
	(0.062)
Birth Cohort: 1980-2000	0.372***
	(0.102)
Birth Cohort: Before 1928 x Women	-0.444***
	(0.083)
Birth Cohort: 1928-1945 x Women	-0.124
	(0.067)
Birth Cohort: 1966-1979 x Women	-0.035
	(0.082)
Birth Cohort: 1980-2000 x Women	0.026
	(0.136)
Region: Midwest	-0.130*
	(0.059)
Region: South	-0.449***
	(0.058)
Region: Pacific	-0.245***
	(0.062)
Region: Midwest x Women	-0.097
	(0.078)
Region: South x Women	-0.042
	(0.076)
Region: Pacific x Women	0.041
	(0.082)
Marital status: Never married	0.360***
	(0.055)
Marital status: Divorced	0.092
	(0.057)
Marital status: Widowed	0.301**
	(0.095)
Marital status: Never married x Women	0.113
	(0.077)
Marital status: Divorced x Women	0.256***
	(0.075)

Table S7. (continued)

	Figure 4 Model
Marital status: Widowed x Women	0.057
	(0.111)
Race: Black (any)	2.566***
	(0.098)
Race: Hispanic (non-black)	0.989***
	(0.090)
Race: All other	0.847***
	(0.146)
Race: Black (any) x Women	0.301*
	(0.129)
Race: Hispanic (non-black) x Women	-0.116
	(0.120)
Race: All other x Women	0.073
	(0.194)
Size-adjusted income quintile	-0.097***
	(0.019)
Women x Size-adjusted income quintile	0.062**
	(0.023)
Constant	-0.183*
	(0.092)
Observations	32709

Source: GSS 1982-2018.

Standard errors in parentheses

* p<0.05, ** p<0.01, *** p<0.001 (two-sided tests)

Table S8. Coefficients from the Models in Figure 5.
All models are logistic regressions that interact each variable (other than gender) with gender. Model 1c is a baseline model that controls for birth cohort and region. Model 2c adds race. Model 3c adds log base 2 household income divided by the square root of the number of people living in the household.

	Model 1c	Model 2c	Model 3c
Women	0.572***	0.658***	0.241
	(0.148)	(0.155)	(0.232)
Birth Cohort: Before 1928	0.399***	0.536***	0.482***
	(0.061)	(0.064)	(0.065)
Birth Cohort: 1928-1945	0.142**	0.194***	0.187***
	(0.048)	(0.050)	(0.050)
Birth Cohort: 1966-1979	0.029	-0.019	-0.036
	(0.059)	(0.063)	(0.064)
Birth Cohort: 1980-2000	0.231*	0.135	0.095
	(0.101)	(0.110)	(0.110)
Birth Cohort: Before 1928 x Women	-0.476***	-0.437***	-0.401***
	(0.083)	(0.088)	(0.088)
Birth Cohort: 1928-1945 x Women	-0.143*	-0.126	-0.121
	(0.064)	(0.068)	(0.068)
Birth Cohort: 1966-1979 x Women	-0.046	-0.078	-0.070
	(0.079)	(0.085)	(0.085)
Birth Cohort: 1980-2000 x Women	-0.052	-0.008	0.013
	(0.137)	(0.146)	(0.146)
Region: Midwest	-0.124*	-0.100	-0.130*
	(0.056)	(0.058)	(0.059)
Region: South	-0.221***	-0.430***	-0.462***
	(0.054)	(0.057)	(0.058)
Region: Pacific	-0.213***	-0.226***	-0.246***
	(0.059)	(0.062)	(0.062)
Region: Midwest x Women	-0.077	-0.110	-0.088
	(0.074)	(0.078)	(0.078)
Region: South x Women	-0.030	-0.074	-0.052
	(0.071)	(0.076)	(0.077)
Region: Pacific x Women	-0.046	0.015	0.033
	(0.079)	(0.082)	(0.083)
Marital Status: Never Married	0.540***	0.487***	0.452***
	(0.052)	(0.055)	(0.056)
Marital Status: Divorced	0.304***	0.139*	0.091
	(0.053)	(0.057)	(0.058)
Marital Status: Widowed	0.426***	0.295**	0.237*
	(0.089)	(0.096)	(0.097)
Marital Status: Never Married x Women	0.296***	0.073	0.090
	(0.072)	(0.077)	(0.078)

Table S8. (continued)

	Model 1c	Model 2c	Model 3c
Marital status: Divorced x Women	0.271***	0.221**	0.240**
	(0.068)	(0.074)	(0.076)
Marital Status: Widowed x Women	0.037	-0.000	0.031
	(0.104)	(0.112)	(0.113)
1980 Election	-0.112	-0.008	-0.059
	(0.116)	(0.121)	(0.122)
1984 Election	-0.369**	-0.310**	-0.354**
	(0.112)	(0.117)	(0.117)
1988 Election	-0.294*	-0.195	-0.230
	(0.116)	(0.121)	(0.122)
1992 Election	-0.119	-0.016	-0.048
	(0.107)	(0.111)	(0.112)
1996 Election	0.120	0.225*	0.205
	(0.110)	(0.114)	(0.115)
2000 Election	-0.237*	-0.178	-0.189
	(0.112)	(0.116)	(0.117)
2004 Election	0.047	0.110	0.109
	(0.112)	(0.117)	(0.117)
2008 Election	0.460***	0.499***	0.494***
	(0.120)	(0.126)	(0.126)
2012 Election	0.467***	0.514***	0.514***
	(0.111)	(0.115)	(0.116)
1980 Election x Women	-0.206	-0.273	-0.251
	(0.158)	(0.166)	(0.167)
1984 Election x Women	-0.303*	-0.392*	-0.377*
	(0.154)	(0.161)	(0.162)
1988 Election x Women	-0.276	-0.385*	-0.372*
	(0.158)	(0.167)	(0.168)
1992 Election x Women	-0.158	-0.245	-0.233
	(0.147)	(0.153)	(0.154)
1996 Election x Women	-0.041	-0.100	-0.094
	(0.152)	(0.158)	(0.159)
2000 Election x Women	-0.096	-0.143	-0.140
	(0.153)	(0.160)	(0.161)
2004 Election x Women	-0.360*	-0.418**	-0.426**
	(0.152)	(0.160)	(0.161)
2008 Election x Women	-0.264	-0.290	-0.294
	(0.163)	(0.171)	(0.172)
2012 Election x Women	-0.259	-0.324*	-0.331*
	(0.154)	(0.160)	(0.161)

Table S8. (continued)

	Model 1c	Model 2c	Model 3c
Race: Black (any)		2.627***	2.567***
		(0.098)	(0.098)
Race: Hispanic (non-black)		1.002***	0.964***
		(0.090)	(0.090)
Race: All other		0.805***	0.804***
		(0.157)	(0.156)
Race: Black (any) x Women		0.280*	0.311*
		(0.129)	(0.130)
Race: Hispanic (non-black) x Women		-0.099	-0.087
		(0.120)	(0.120)
Race: All other x Women		0.080	0.073
		(0.203)	(0.202)
Size-adjusted household income			-0.109***
			(0.023)
Women x Size-adjusted household income			0.067*
			(0.028)
Constant	-0.354**	-0.642***	0.036
	(0.108)	(0.112)	(0.181)
Observations	32730	32730	32709

Source: GSS 1982-2018.

Standard errors in parentheses

* $p<0.05$, ** $p<0.01$, *** $p<0.001$ (two-sided tests)

Replication in the ANES. The remaining figures in this SI Appendix present our replication of our main GSS findings using the American National Election Study (ANES). Analogous to Figure 1 in the paper, Figure S2 shows the percent of men and women in the ANES voting Democratic in each election. Like in the GSS, the ANES does not find any nontrivial amount of the gender gap explained by income in any of Figures S3-S5. (In this ANES analysis we transformed household income to base 2 log, as we did for the GSS, but, since the ANES did not ask about household size, we could not size-standardize the measure as we did in the GSS analysis.)

Like the GSS analysis, the ANES analysis also suggests that the different race composition of men and women voters explains part of the gender gap. The mediation of the gender gap by race escapes statistical significance in the ANES, but the patterns shown in the GSS and ANES are strikingly similar. Figure S3, which pools across all marital statuses and years, shows that, controlling for race reduces the gender gap, as it did in Figure 2 in the main paper. Showing separate results by marital status but pooling years, similar to the GSS findings in Figure 3 in the main paper, Figure S4 shows that controlling for race does not change the gender gap among the married, but that the estimated gender gaps among never-married and divorced voters are reduced when race is controlled. Figure S5 shows the proportion voting Democratic separately by race, gender, marital status, and income quintile; it is parallel to Figure 4 in the paper. It illustrates why income does not explain why women voter more Democratic than men; the effect of income, while present for white men, is very small compared to the effect of race, and is barely discernible for any group other than white men. Figure S6 is parallel to Figure 5 in the paper; it shows gender gaps by marital status and election year, making clear that the pattern of race explaining some of the gender gap among unmarried voters is true for most years, whereas race explains none of the gender gap among the married in any year, and income explains none of the gender gap in any year.

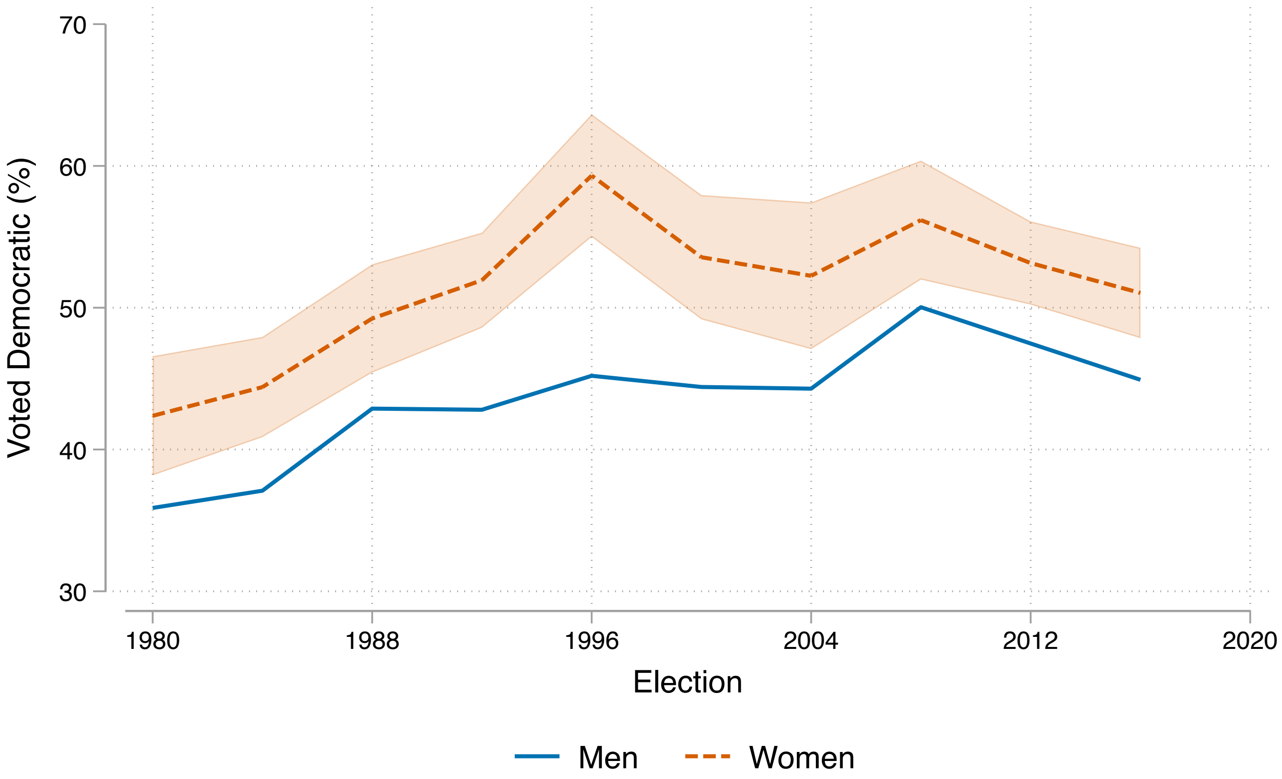

Fig. S3. Percentage Voting for the Democrat in Presidential Elections by Election and Gender, United States, 1980–2016
Source: American National Election Studies Cumulative Data File, 2018.

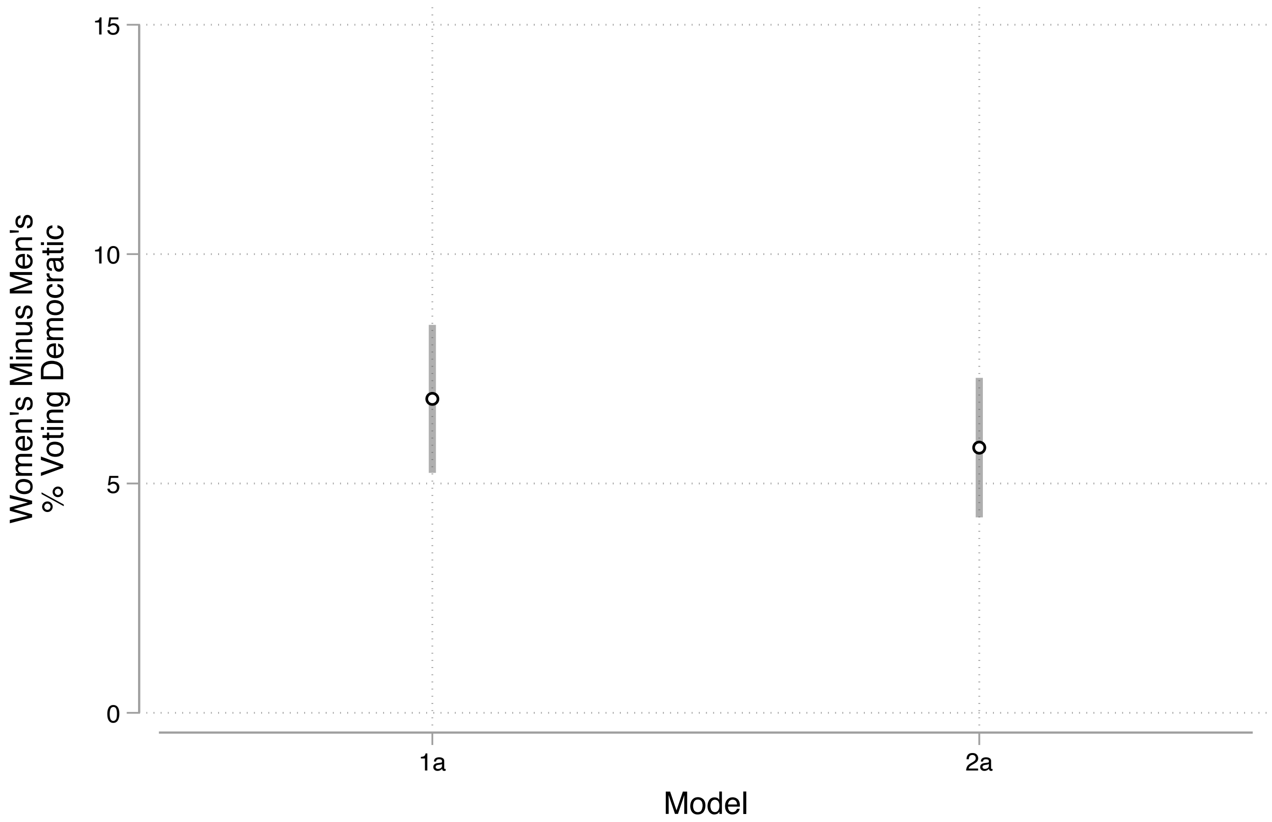

Fig. S4. Marginal Gender Difference in Voting for the Democrat in Presidential Elections by Model, United States, 1980–2016
Notes: **Model 1** includes gender, election year, cohort, and region; **Model 2** adds race. See text for details about interactions among variables.
Source: American National Election Studies Cumulative Data File, 2018.

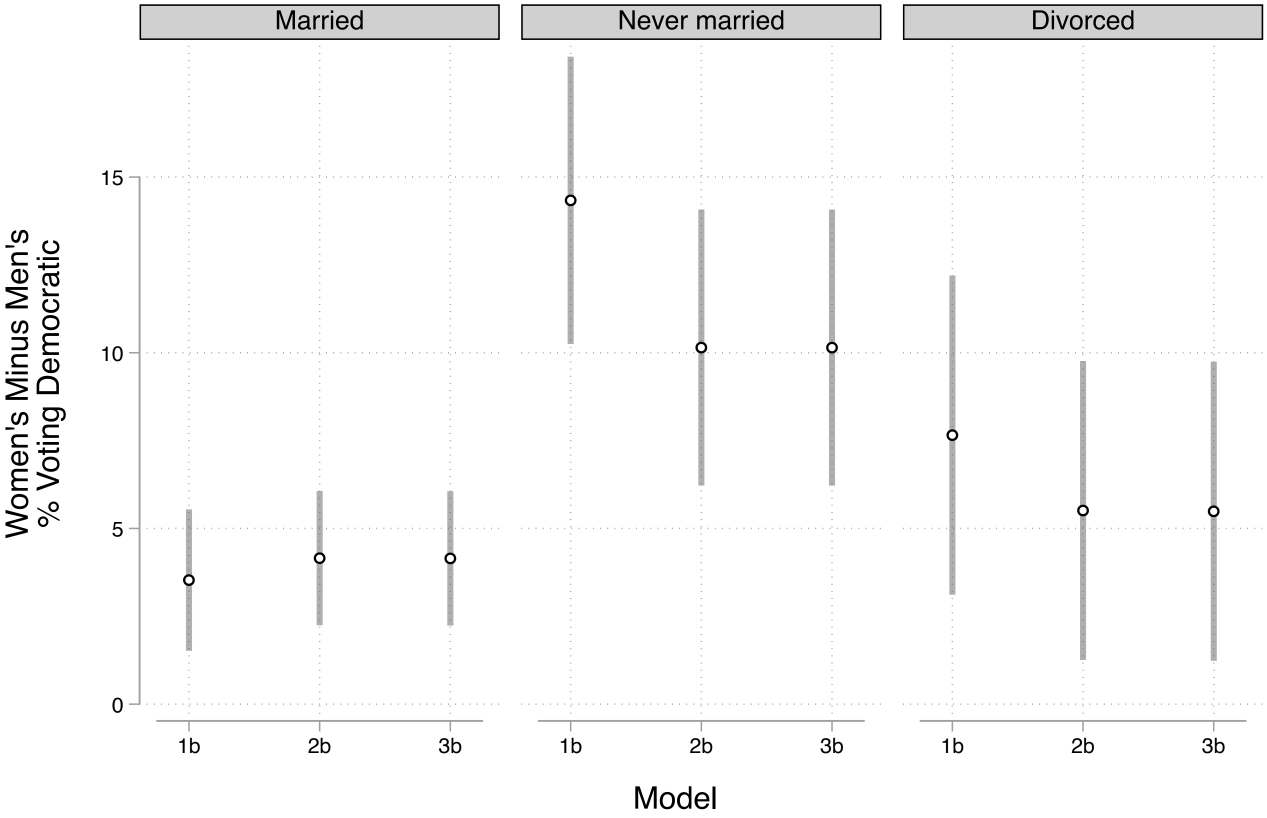

Fig. S5. Marginal Gender Difference in Voting for the Democrat in Presidential Elections by Model and Marital Status, United States, 1980–2016
Notes: **Model 1** includes gender, election year, cohort, and region; **Model 2** adds race; **Model 3** adds household income. See text for details about interactions among variables.
Source: American National Election Studies Cumulative Data File, 2018.

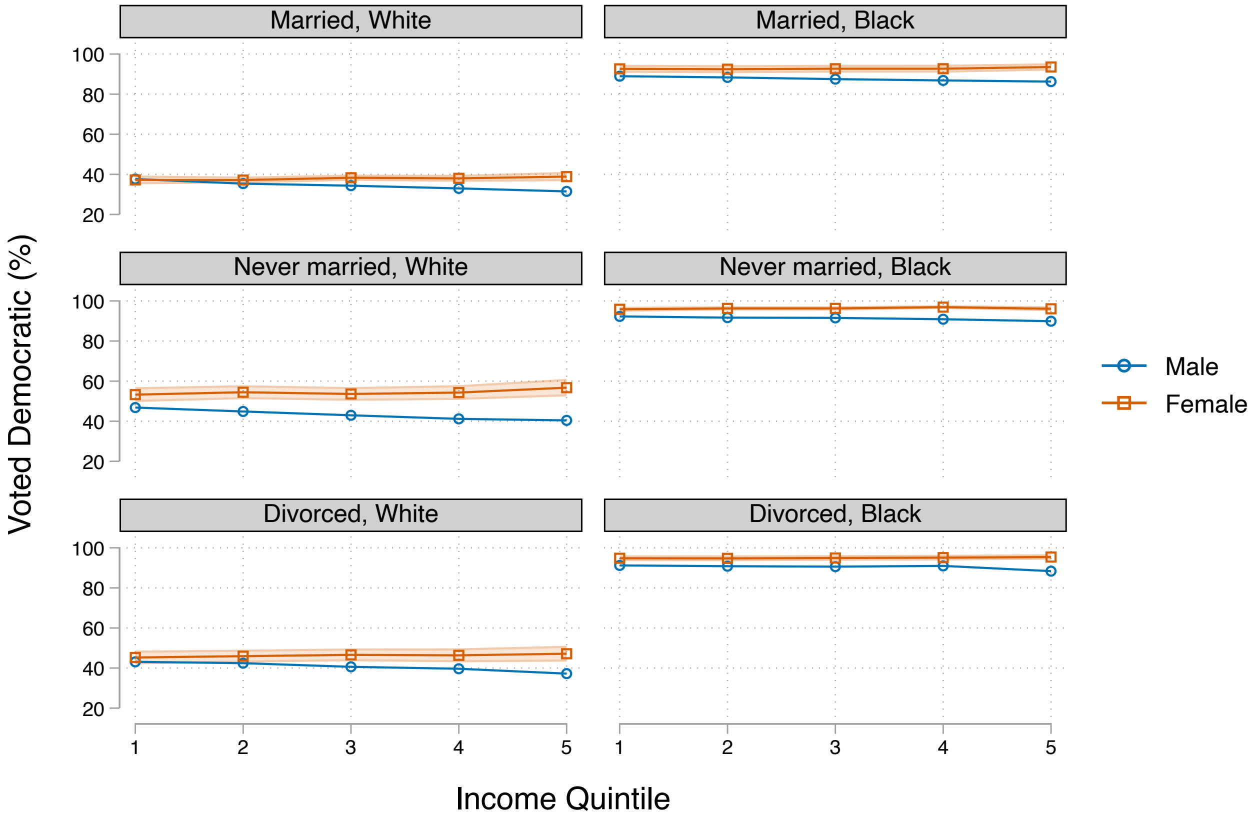

Fig. S6. Marginal Probability of Voting Democratic Across Income Quintiles by Gender, Race and Marital Status, 1980-2016
Notes: Estimates based on Model 3b with the continuous income measure converted to quintiles.
Source: American National Election Studies Cumulative Data File, 2018.

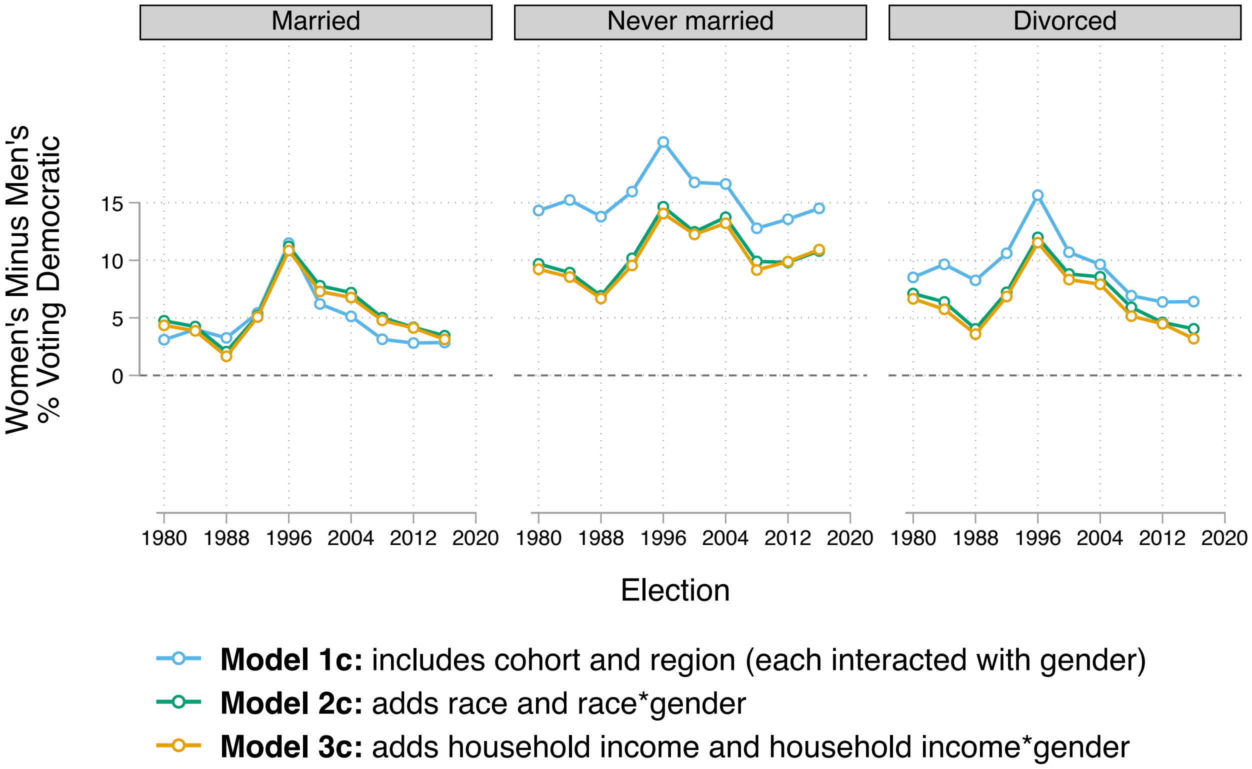

Fig. S7. Marginal Gender Difference in Voting for the Democrat in Presidential Elections by Election, Model and Marital Status, United States, 1980–2016
Notes: See text for details about interactions among variables.
Source: American National Election Studies Cumulative Data File, 2018.
